# Techno-economic process modelling and Monte Carlo simulation data of uncertainty quantification in field-grown plant-based manufacturing

**DOI:** 10.1016/j.dib.2021.107317

**Published:** 2021-08-21

**Authors:** Matthew J. McNulty, Kirolos Kelada, Debashis Paul, Somen Nandi, Karen A. McDonald

**Affiliations:** a Department of Chemical Engineering, University of California, Davis, CA, USA; b Department of Statistics, University of California, Davis, CA, USA; c Global HealthShare Initiative, University of California, Davis, CA, USA

**Keywords:** Plant molecular farming, Plant-based manufacturing, Uncertainty quantification, Process simulation tool, Techno-economic analysis, Agricultural production

## Abstract

This data article is related to the research article, **“**M.J. McNulty, K. Kelada, D. Paul, S. Nandi, and K.A. McDonald, Introducing uncertainty quantification to techno-economic models of manufacturing field-grown plant-made products, Food Bioprod. Process. 128 (2021) 153–165.” The raw and analyzed data presented are related to generation, analysis, and optimization of ultra-large-scale field-grown plant-based manufacturing of high-value recombinant protein under uncertainty. The data have been acquired using deterministic techno-economic process model simulation in SuperPro Designer integrated with stochastic Monte Carlo-based simulation in Microsoft Excel using the Crystal Ball plug-in. The purpose of the article is to make techno-economic and associated uncertainty data available to be leveraged and adapted for other research purposes.

## Specifications Table

**Table:** 

Subject	Chemical Engineering: Process Chemistry and Technology
Specific subject area	Process Engineering
Type of data	Table Chart Graph
How data were acquired	Process simulation tool SuperPro Designer® version 10 build 7 and Microsoft Excel with the Oracle® Crystall Ball plugin.
Data format	Raw and analyzed model simulation output data.
Parameters for data collection	Data are values collected from process simulation input parameter and forecast variables selected based on working process knowledge. • Input parameters: field growth yield, field growth time, expression level, harvesting time, plate & frame filtration recovery and flux, tangential flow filtration recovery and flux, chromatography recovery. • Forecast variables: internal rate of return (after tax), cost of goods sold, annual throughput, product purity
Description of data collection	Data are collected directly from software tools.
Data source location	N/A
Data accessibility	Repository name: Mendeley Data Data identification number: 10.17632/h5s7rz29vg.1 Direct URL to data: http://dx.doi.org/10.17632/h5s7rz29vg.1
Related research article	M.J. McNulty, K. Kelada, D. Paul, S. Nandi, and K.A. McDonald, Introducing uncertainty quantification to techno-economic models of manufacturing field-grown plant-made products, Food Bioprod. Process. 128 (2021) 153–165. [1]

## Value of the Data

• The first reported data of uncertainty quantification in techno-economic models of plant-based bioproducts manufacturing.
• The generated data is produced for a generic bioproduct and can be used by parties in development of their specific bioproduct manufacturing strategies.
• It provides valuable insights into approaching uncertainty in ultra-large-scale field-grown plant-based manufacturing and serves as a guideline for future approaches.

## 1 Data Description

The data are from techno-economic uncertainty quantification of field-grown plant-based manufacturing of a generic food-grade bioproduct [1]. The data can be grouped as follows:

### 1.1 Generation of techno-economic data under uncertainty

The input parameter assumption distributions and associated Monte Carlo sampling-based trial data for the base case techno-economic process model are described in the data file, *0.1 Assumption distribution & trial data (base case)*. These assumptions distribution trial data feed into the techno-economic process model (publicly available at http://mcdonald-nandi.ech.ucdavis.edu/tools/techno-economics/) to generate the forecast variable output data in the base case scenario and facility oversizing scenarios (in which the equipment of the facility is sized larger to accommodate the uncertainty of production). This is described in data file, *02. Simulation trial data (base case + facility oversizing).* The details of the equipment oversizing scenarios of the techno-economic process model to accommodate the uncertainty of production are described in the data file, *03. Equipment oversizing specification (base case + facility oversizing)*.

### 1.2 Analysis of techno-economic forecast variable outputs

The forecast variable output data are compared between the base case and facility oversizing scenarios using two-sample t-tests for evaluation of the means and Kolmogorv-Smirnov tests for evaluation of the distributions, which is summarized in the data file, *04. Statistical test results (base case + facility oversizing)*. Box plots and quantile-quantile plots are shown in the data file, *05. Forecast variable normality (base case + facility oversizing)*, as assessments of normality. Univariate sensitivity of the forecast variables to the input parameters is investigated using tornado plots and spider charts in the data files, *06. Forecast univariate sensitivity data (base case)* and *07. Forecast univariate sensitivity charts (base case)*. The contribution to variance of each input parameter to each forecast variable is calculated by rank correlation coefficient using Monte Carlo-based techno-economic simulation run data for the base case in which Pearson correlation coefficients are not included, the results of which are described in the data file, *08. Contribution to variance (base case)*. Techno-economic output metrics (e.g., cost breakdown by section and cost item, total capital expenditures, number of batches per year) are generated in the techno-economic modelling software using input parameter values associated with Monte Carlo-based techno-economic simulation trials that yielded the minimum, mean, and maximum values of internal rate of return after tax for the base case and facility oversizing scenarios, as described in the data file, *09. Cost breakdowns (base case + facility oversizing)*.

### 1.3 Techno-economic optimization under uncertainty

A facility retrofitting case which presumes that the cation exchange chromatography is a new addition to an existing facility is approached with the base case facility sizing assumed to be fixed and the cation exchange chromatography column diameter is set as a decision variable to minimize internal rate of return after tax, as described in the data file, *10. Simulation results summary (CEX size optimization)*.

## 2 Experimental Design, Materials and Methods

The method used to generate the foundational data presented in this article is an integration of a deterministic techno-economic process model simulation in SuperPro Designer with Monte Carlo-based stochastic simulation of input parameter uncertainty using assumption distributions and Pearson correlation coefficients supported by literature and working process knowledge in Microsoft Excel using the Crystal Ball plug-in.

### 2.1 Assessment of assumption distributions

Assumption distributions were primarily determined by working process knowledge supported by reports in literature. We used our working process knowledge to select probability distributions reflective of plant-made pharmaceutical production that one might observe at lab- and/or pilot-scale production. The probability distributions are not based on any existing commercial facility capability.

Expression level variations were performed by changing the mass coefficients of “Product” and “Biomass week 6” in P-18’s RXNSEP-1, while keeping their sum constant. The probability distribution profile was obtained from Werner et al. [2]. The data were normalized so that the mean is 1.50 g/kg (base case expression level) and was best fit by a logistic distribution. A triangular distribution was used to represent the uncertainty with field growth time before induction in P-16. Mechanical harvesting (P-21/GBX-104) time variability was represented by a beta distribution with minimum and maximum values based off an assumed 1–10 km/h harvester speed. UF/DF filtrate flux was assumed to vary by ±25% from the base case value according to a triangular distribution [3]. Cation exchange chromatography (CEX) losses were assumed to vary according to a uniform distribution with minimum and maximum values ±10% their mean (base case) [4]. Harvesting time, plate & flame removal, and field growth time distributions were based on assumptions determined using working process knowledge not directly supported by reported values in public literature.

The field growth yield probability distribution was derived from an analysis of previously published literature. The following Sections 2.1.1–2.1.2 detail the development of the field growth yield assumption distribution.

#### 2.1.1 Model for tobacco dry weight estimation as a function of temperature

Experimental data of tobacco dry weight were extracted from Fig. 1 (A–E) in [5] using an open source software DataTheif (B. Tummers, DataThief III. 2006 https://datathief.org/). The data was collected by measuring the dry weight of plants at different intervals during their growth; the high and low temperatures were kept constant for 9 and 15 h, respectively. A weighted average of these temperatures was used in the model calculations (i.e., 9/24*high + 15/24*low). Plants were rotated between different growth chambers where high and temperatures were kept constant, however, different in each chamber, resulting in 10 different sets of experimental data. To construct the model, all 10 sets of data were fit to Eqs. (2) and (3) by using an initial guess for the equation parameters (A,k,S,H) from Wann et al. [6] and calculating the root mean squared error (RMSE) - (Eq. (3)). A built-in Microsoft-Excel solver was then used to find the model parameters that would minimize the RMSE between model predictions and experimental data.

(1) $$W_{i}=W_{0}+\left(W_{0}\cdot r\left(T\right)\cdot \Delta t\right)$$

**Fig. 1:**
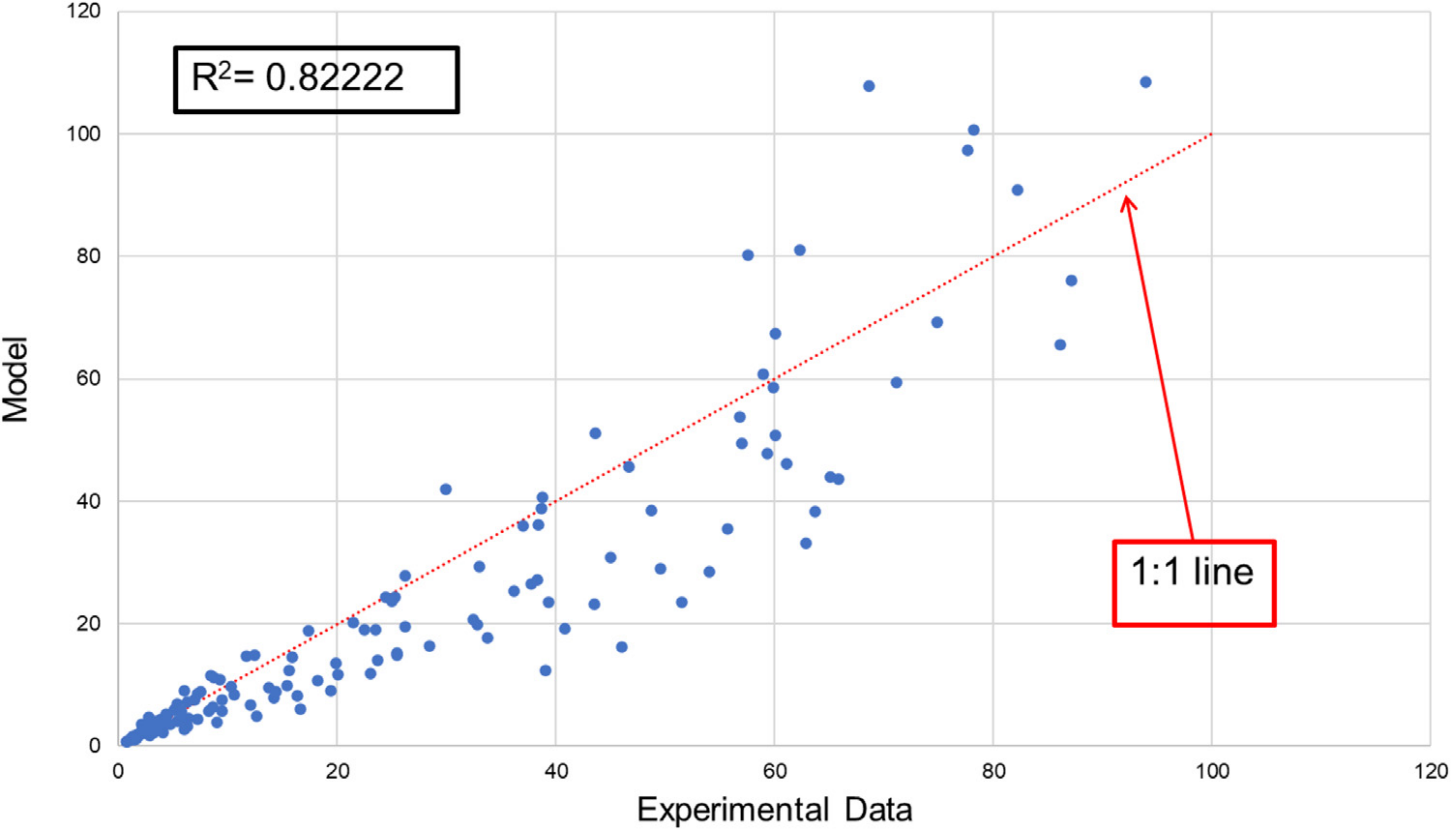
Regression plot of model vs. experimental data points, showing calculated *R*^2^ value.

Where W_i_ – tobacco dry weight at day “*i*” post-transplant (g/plant), *W_0_* – initial dry weight at day “*i*-Δt”(g/plant), r(*T*) temperature- dependent growth rate in g/(plant day), Δt – time between *W_0_* and *W_i_* in days.

(2) $$r\left(T\right)=\frac{A\;T\;\text{exp}\left(\frac{-k}{T}\right)}{1\;+\exp\left(S-\frac{H}{T}\right)}$$

A,k,S,H – model parameters, *T* – temperature (K).

There is an optimum temperature for tobacco growth below which the growth rate follows the Arrhenius law. Above this optimum temperature, the rate declines due to the inactivation of enzymes and the denaturation of plant proteins. Therefore, the complex function (Eq. (2)) was chosen to model the growth rate response to temperature [6].

(3) $$RMSE\;=\sqrt{\frac{1}{N}\;\sum {\left(y_{i}-o_{i}\right)}^{2}}$$

Where *y_i_* is model prediction, *o_i_* is experimental data, and *N* is the total number of predictions/observations.

All data points – experimental on x-axis and model predictions on y-axis– were plotted on the same graph (Fig. 1), in addition to a 1:1 line to show model deviation from experimental data. The *R*^2^ value was calculated using Eq. (4)

(4) $$R^{2}=\;1-\;\frac{\sum {\left(y_{i}-o_{i}\right)}^{2}}{\sum {\left(o_{i}-o_{avg}\right)}^{2}}$$

Where *y_i_* – model prediction, *o_i_* – observation, and *o_avg_* – average of all observations.

**Fig. 2:**
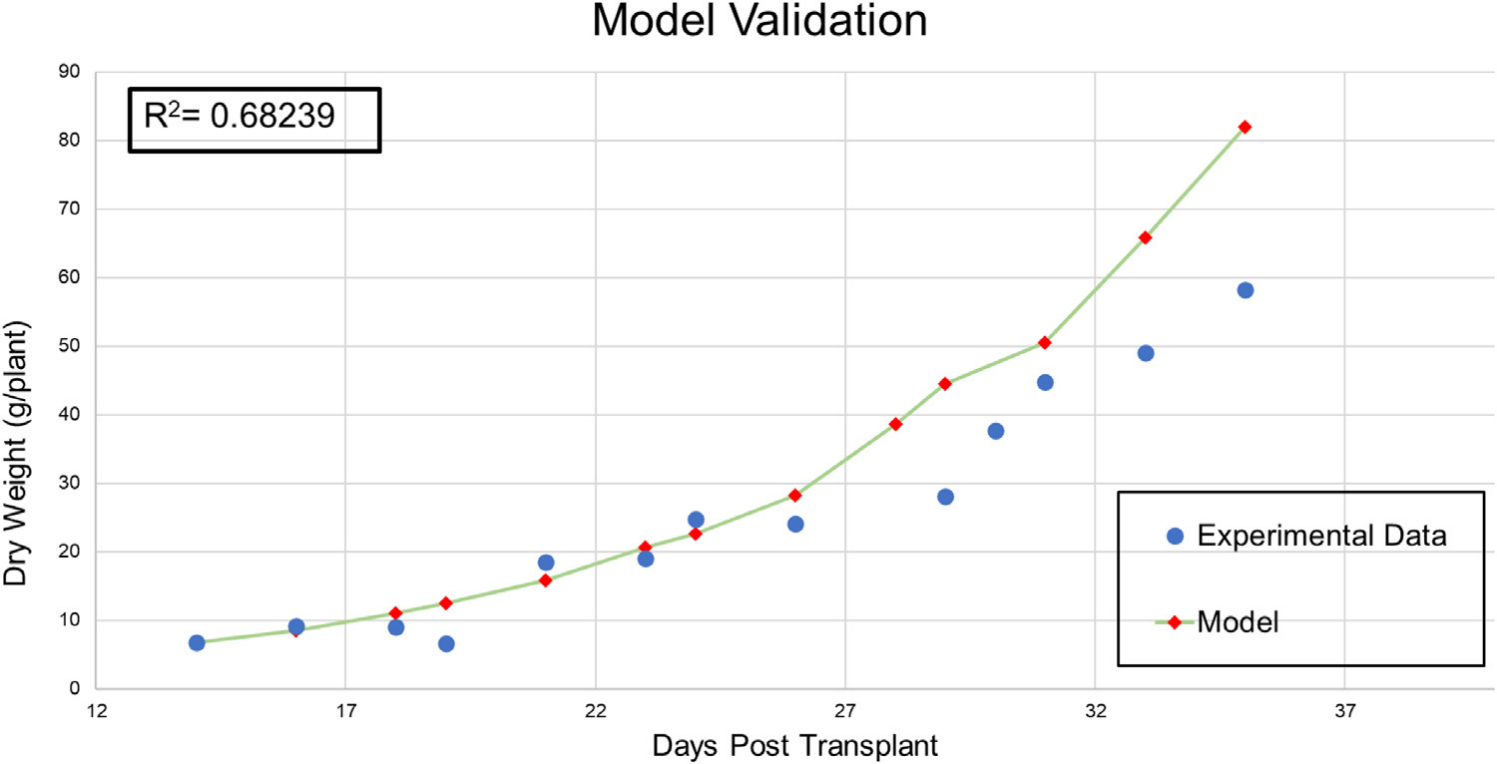
Model validation results using data in Fig. 2 in Wann and Raper [5].

**Fig. 3:**
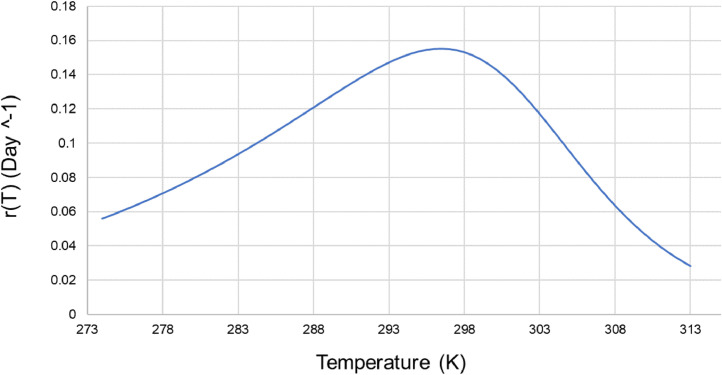
Growth rate as a function of temperature based on fitted model parameters.

Model parameters that result in a minimized RMSE value of 9.839 are shown in Table 1. The model was validated using a different set of experimental data (obtained from Fig. 2 in [5]. The two sets of data were plotted on the same graph (Fig. 2) with the calculated R^2^ value. Fig. 3 shows the growth rate “*r*(*T*)” values for a range of temperature. It confirms previously reported optimal growth range (18.5–28.5 °C) [7] which corresponds to 291.5–301.5 K.

**Table 1:** Model parameters obtained by minimizing the root mean squared error (RMSE).

*A* (day^−1^ K^−1^)	*S*	*k* (K)	*H* (K)
924.6	75.43	4199	22,780

#### 2.1.2 Tobacco yield estimation and monthly variations

The previous model was used to predict tobacco dry weight per plant as a function of temperature. Hourly temperature (60 cm above ground level) data in Homestead, Florida was obtained from the Florida automated weather network (FAWN) database for three consecutive years (2017–2019). The model predicts tobacco dry weight at day 27 after emergence of seedlings (assuming a constant initial dry weight of 0.5 g/plant seedling), starting at the first hour of the first day of every month and ending on the 23rd hour of the 27th day of the same month. Assuming that germination occurs over the course of 15 days, the model predicts the dry weight yield at day 42 post seeding.

**Fig. 4:**
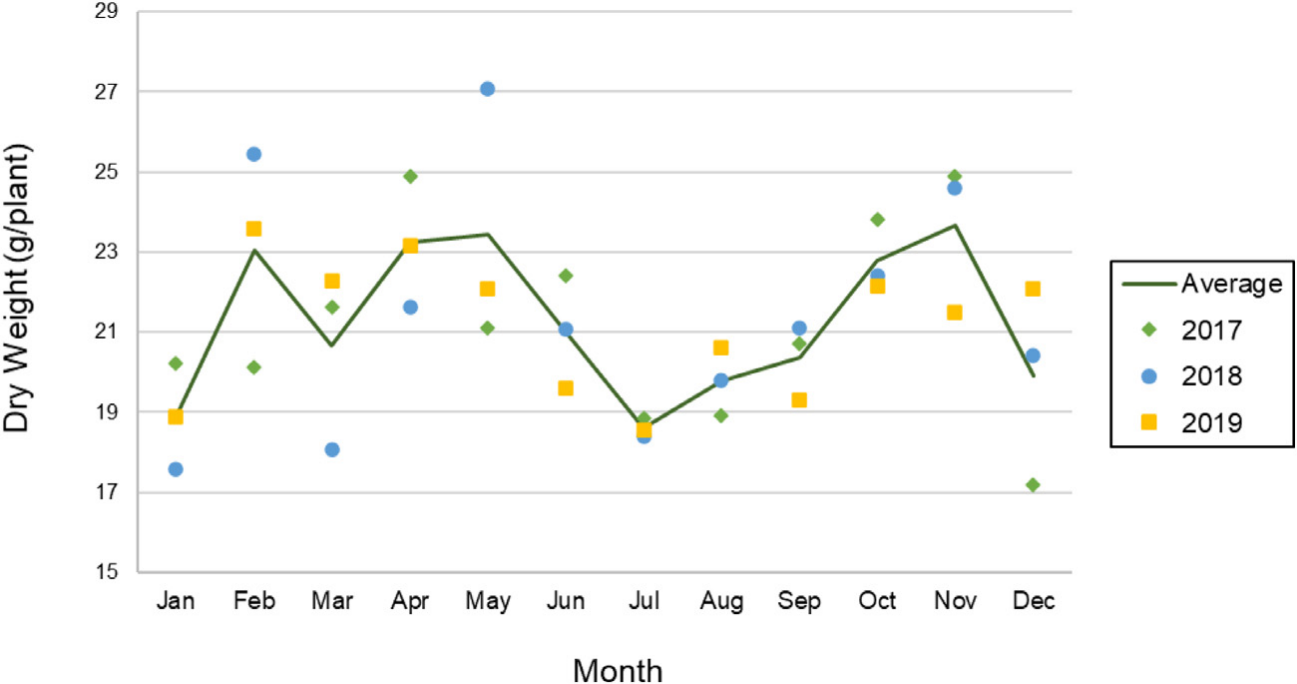
Tobacco dry weight prediction based on historical Homestead, FL weather data from three consecutive years. (For interpretation of the references to color in this figure, the reader is referred to the web version of this article).

Fig. 4 shows the model results for each year at the end of 27th day of each month. The average of the monthly yield over 3 years is also displayed as a solid green line, indicating a slight drop in yield during the month of July, most likely due to the consistently elevated temperature. The average dry weight yield was 21.28 ± 2.37 g/plant. This 11% standard deviation from the mean reflect a low variation in temperature ranges due to seasonal changes in Homestead, Florida. However, this model can be further improved by incorporating other weather factors such as photon flux, ambient CO_2_ concentration, plant nutrients availability, wind, and humidity. Germination efficiency as a function of these variables should also be considered to produce a more robust model.

#### 2.1.3 Probability distribution justification

The data generated from the dry weight yield prediction model (*n* = 36) was used to obtain biomass conversion distribution, assuming a linear relationship between fresh weight and dry weight. The yield prediction model results were normalized by its maximum value and were best fit by a beta probability distribution (alpha = 2.57, beta = 4.80, minimum = 0.63, maximum = 1). The mean of this distribution was calculated to be 0.76 (base case).

### 2.2 Techno-economic process model simulation

The data described here and in the associated research article builds on the techno-economic design bases and assumptions previously established in Kelada et al. [8]. The SuperPro Designer model used in this methodology has been modified to produce a generic high-value recombinant protein and for compatibility with uncertainty quantification. Namely, there are two significant changes for compatibility with uncertainty quantification: (1) upstream and downstream processing models have been merged so that input parameter variations simply propagate throughout the entirety of the facility simulation, and (2) equipment size has been fixed according to the static base case values such that uncertainty is largely absorbed by the rated throughput of the equipment. Uncertainty in stream volume and product mass per batch cannot be entirely absorbed by the facility model and so simple algorithmic fixes were implemented for the field growth (P-2) and cation exchange (P-20) procedures such that yield and recovery, respectively, as reduced to “effective” values corresponding to maximal stream volume and product mass per batch, respectively, in the cases when excess stream volume or product mass per batch arise stochastically from the input parameter uncertainty values.

### 2.3 Monte Carlo simulation

Monte Carlo simulation used to generate the data described here is performed using Microsoft Excel with the Crystal Ball plug-in, which allows the user to simply define probability distribution assumptions, correlations between assumptions, forecast variables, and decision variables to any number of spreadsheet cells as well as to run Monte Carlo simulation in Excel using those definitions. Additionally, Crystal Ball's built-in OptQuest is used to generate the data for the optimization scenario. Simulations are executed using 20,000 trials for each of the three selling prices analyzed, meaning that profitability-related forecast variables are analyzed using data from 20,000 trials while process-related forecast variables are analyzed using data from 60,000 trials (combined data from each of the selling prices).

### 2.4 Simulation integration

The techno-economic process model simulation and the Monte Carlo simulation are integrated in a master-slave relationship using custom Visual Basic for Application scripts in Microsoft Excel to interact with SuperPro Designer via the built-in Component Object Module library to set the techno-economic process model with stochastically-generated input parameter values from Monte Carlo simulation, execute mass and energy balances and economic calculations in the techno-economic process model simulation, and to record the updated forecast variable outputs from the techno-economic process model simulation in Excel for each Monte Carlo simulation trial.

## Ethics Statement

The authors followed generally expected standards of ethical behavior in scientific publishing throughout article construction.

## CRediT Author Statement

**Matthew J. McNulty:** Conceptualization, Methodology, Formal analysis, Visualization, Writing – original draft; **Kirolos Kelada:** Conceptualization, Methodology, Writing – original draft; **Debashis Paul:** Supervision, Writing – review & editing; **Somen Nandi:** Supervision, Writing – review & editing; **Karen A. McDonald:** Supervision, Writing – review & editing.

## Declaration of Competing Interest

The authors declare that they have no known competing financial interests or personal relationships which have or could be perceived to have influenced the work reported in this article.
